# Administration of mulberry leaves maintains pancreatic β-cell mass in obese/type 2 diabetes mellitus mouse model

**DOI:** 10.1186/s12906-020-02933-4

**Published:** 2020-05-06

**Authors:** Patlada Suthamwong, Manabu Minami, Toshiaki Okada, Nonomi Shiwaku, Mai Uesugi, Masayuki Yokode, Kaeko Kamei

**Affiliations:** 1 Department of Functional Chemistry, Kyoto Institute of Technology, Matsugasaki, Sakyo-ku, Kyoto, 606-8585 Japan; 2 Department of Clinical Innovative Medicine, Kyoto University Graduate School of Medicine, 54 Kawahara-cho, Shogoin, Sakyo-ku, Kyoto, 606-8507 Japan

**Keywords:** Mulberry leaves, β-Cell, Endoplasmic reticulum stress, Obesity, Type 2 diabetes

## Abstract

**Background:**

Type 2 diabetes mellitus is characterized by insulin resistance and pancreatic β-cell dysfunction. A decrease in β-cell mass, which occurs during the progression of Type 2 diabetes mellitus, contributes to impaired insulin secretion. Mulberry leaves contain various nutritional components that exert anti-diabetic and anti-atherogenic effects. The present study analyzed the effects of mulberry leaf intake on pancreatic β-cells to clarify the mechanisms underlying its anti-diabetic function.

**Methods:**

Mulberry leaves (*Morus alba* L.) were dried at 180 °C for 8 s in a hot-air mill and fed to obesity/Type 2 diabetes mellitus db/db mouse models at 5% (w/w) as part of a normal diet from 7 to 10, 15, or 20 weeks of age. An intraperitoneal glucose tolerance test was then performed on the mice. To evaluate the β-cell mass, the pancreas was subjected to immunohistological analysis with an anti-insulin antibody. A TUNEL assay and immunohistological analysis with a proliferation marker was also performed. Expression levels of endoplasmic reticulum stress-responsible genes and proliferation markers were assessed by quantitative RT-PCR.

**Results:**

Intake of mulberry leaves maintained the β-cell function of db/db mice. Moreover, oral administration of mulberry leaves significantly decreased cell death by reducing endoplasmic reticulum stress in the pancreas. Mulberry leaves significantly increased proliferation of β-cells and the expression of *pancreatic duodenal homeobox1* mRNA in the pancreas.

**Conclusion:**

Considered together, these results indicate that dietary mulberry leaf administration can maintain insulin levels and pancreatic β-cell mass, at least in part, by suppressing endoplasmic reticulum stress in Type 2 diabetes mellitus mouse models.

## Background

Obesity is considered a critical issue worldwide. It is known that obesity may lead to certain metabolic disorders such as cardiovascular disease and diabetes mellitus [1]. The number of diabetic patients is estimated to be over 400 million, globally [2]. Type 2 diabetes mellitus (T2DM) is characterized by insulin resistance and the dysfunction of insulin producing β-cells in the islets of Langerhans of the pancreas [3, 4]. Many studies have indicated that insulin resistance precedes hyperglycemia, which eventually leads to T2DM [5–7]. During the progression of insulin resistance, decreased glucose-intake in adipose tissues and muscles, compounded by enhanced gluconeogenesis and glycogenolysis in the liver, may cause chronic high blood glucose levels leading to increased insulin demand. Weir et al. classifies progression in two stages. Stage 1 is the compensation stage, which occurs during diabetes progression, whereby β-cell adaptation increases in the β-cell area in the pancreas in order to upregulate insulin secretion in response to the increased demand for insulin. Subsequently, stage 2 is the decompensation stage, when the β-cell area decreases due to activation of cell death by higher endoplasmic reticulum (ER) stress and oxidative stress conditions [8, 9].

Recent studies have indicated that unfolded proteins are important factors that cause insulin resistance by stimulating ER stress. Unfolded proteins are present in white adipose tissue, pancreas, liver, and skeletal muscles of insulin resistant rodents [10]. In the ER lumen, a HSP70 molecular chaperone, immunoglobulin heavy-chain binding protein (BiP), also called glucose related protein 78 (GRP78), plays a role in the folding of unfolded proteins. Its activity is regulated by binding with an ER stress sensor protein, inositol requiring enzyme 1 (IRE1), PKR like endoplasmic reticulum kinase (PERK) or activating transcription factor 6 (ATF6) [11]. During unfolded protein response (UPR), BiP dissociates from IRE1, PERK, or ATF6, and binds to unfolded proteins [8], thereby activating the ER stress sensor protein to promote the translocation of factors such as activating transcription factor 4 (ATF4) and x-box binding protein 1 (XBP1). These factors induce the expression of ER chaperon proteins and pro-apoptosis proteins, including the C/EBP homologous protein (CHOP) [12]. Excess ER stress leads to the accumulation of unfolded proteins within the ER, eventually causing cell death. Pancreatic β-cells, in particular, produce and secrete insulin to regulate glucose concentration in plasma following a meal or hormone release, thereby inducing the reuptake of glucose by cells. Increased secretion of insulin under hyperglycemic conditions may overwhelm the capacity of the ER, causing ER dysfunction. Many studies have revealed that in vivo deletion of *Chop* or *Atf4* knockout in Akita spontaneous diabetes mouse models, not only protected β-cells from cell apoptosis, but also improved protein folding in the ER [13]. Other reports indicated that *Xbp1* deficiency caused hyperglycemia and glucose intolerance in mice [14]. The deficiency of p85α, a regulatory subunit of phosphatidylinositol-3-kinase (PI3K), in Akita mice reduced ER stress and the protein expression level of Xbp1, a transcription factor involved in UPR in β-cells, thus delaying activation of the apoptotic pathway [15].

Intake of mulberry leaves (ML) (*Morus alba* L; *Moraceae*), commonly used as a diet for silkworms (*Bombyx mori* L.), exerts beneficial anti-hyperglycemic effects in humans [16], anti-atherogenic effects in mice [17], as well as antioxidant effects. ML is used to treat diabetes in Chinese medicine [6]. Studies conducted by us previously indicated that oral administration of ML ameliorated dysregulation of adipocytokine in the white adipose tissue (WAT) of db/db mouse obesity and T2DM models [18]. ML contains 1-deoxynojirimycin (DNJ), a glucose analog which suppresses postprandial blood glucose levels by inhibiting α-glucosidase. ML also contains a rich antioxidant which may reduce reactive oxygen species (ROS) [19]. We previously reported that administration of ML ameliorated abnormal glucose tolerance and suppressed the expression of NADPH oxidase, a ROS generating enzyme, in the WAT and liver of db/db mice, resulting in the reduction of oxidative stress [18]. Some studies have also described the effects of antioxidants contained in ML. Youl et al., reported that quercetin enhanced insulin secretion and reduced oxidative damage in rat pancreatic islets treated with H_2_O_2_ [20]. Similarly, administration of isoquercetin for 5 weeks lowered blood glucose levels in KK-Ay mouse non-insulin-dependent diabetes models [21]. These flavonoids are known to be components of ML.

We found that oral administration of ML to db/db mouse obesity/T2DM models improved glucose tolerance, indicating an effect of ML on insulin secretion in the pancreas. However, the effects of ML intake on β-cells are yet to be revealed. Thus, the objective of the present study was to investigate the effects of oral ML administration on pancreatic function in db/db mice.

## Methods

### Ethics statement

This study was performed with the approval of the Institutional Animal Care and Use Committees (IACUC), ethics committee of Kyoto University (approval No. MedKyo 19,301). All sections of this report are based on the ARRIVE Guidelines for reporting animal research [22]. The mice were deeply anesthetized with 40 mg/kg of pentobarbital sodium (Kyoritsu Seiyaku, Tokyo, Japan); according to terminal procedures under anesthesia, blood was withdrawn and tissues were collected. All efforts were made to minimize suffering.

### Mulberry leaves

The three races of Mulberry tree (*Morus alba* L.), “Hayate-sakari,” “Ichinose,” and “Minamisakari” had been stocked in the experimental farm of Kyoto Institute of Technology. These were transplanted to Kyotango Furusato Farm (Kyoto, Japan) by planting branches, and cultured using Japanese standard methods. A mixture of mulberry leaves was harvested from the three races “Hayate-sakari,” “Ichinose,” and “Minamisakari” at a ratio of 5:3:2 in the Kyotango Furusato Farm. Mulberry leaf powder was prepared by drying and milling at 180 °C for 8 s in a hot-air mill (Drymeister, Hosokawa Micron, Osaka, Japan). The average diameter of the dried powder used in this experiment was 20 μm.

### Animals and diets

Five-week-old (5-w) male db/db mice and regular chow were purchased from Oriental Bio Service (Kyoto, Kyoto, Japan). The db/db mice were randomly allocated into two diet-groups, control-diet group and ML-diet group. Each diet group was further divided into three groups (each *n* = 6) by feeding duration from 7-w age to 10, 15, or 20-w age. Mice of all diet groups were provided ad libitum access to regular chow diet and water and were acclimated for at least 7 days under conditions of controlled temperature and a 12 h light/dark cycle. From 7-w of age, mice of the control-diet group were fed regular chow diet consisting of 4.8% fat, 61.4% carbohydrate, 20.8% protein, 8.0% water and 5.0% ash, while the ML-diet group were fed regular chow containing 5% (w/w) dried ML. The diets were stored at 4 °C until use. Mice of each diet group were euthanized at 10, 15 and 20 weeks of age, bloods were collected, and pancreas tissues were removed and frozen in liquid nitrogen. All samples were stored at − 80 °C. All procedures were approved by the Kyoto University.

### Blood analysis and glucose tolerance test

All blood samples were collected following an overnight fast. Blood glucose levels and insulin concentrations were measured using the commercial kits, Glucose CII test Wako kit (Mutarotase-GOD method, Wako Pure Chemical Industries, Osaka, Japan) and Morinaga Ultra-Sensitive Mouse/Rat Insulin ELISA kit (MIoBS, Yokohama, Japan), respectively. For the intraperitoneal glucose tolerance test (ipGTT), mice were subjected to fasting for 16 h, and intraperitoneally injected with 1.5 g/kg of D-glucose (Wako). Blood samples were collected before and then sequentially after injection. The ipGTT tests were done on the same day for mice from the same age group of both diet groups.

### Immunohistological analysis

The pancreases were fixed overnight in 4% paraformaldehyde (Wako) and embedded in paraffin. Sections were incubated with primary antibodies overnight at 4 °C. DAPI (diamidino-2-phenylindole) dye (Invitrogen, Carlsbad, CA, USA) was used to detect nuclei in immunofluorescent images. Primary antibodies used were as follows: mouse anti-insulin + proinsulin antibody (Abcam, Cambridge, MA, USA, ab8304, 1:1250), rabbit anti-insulin (Santa Cruz Biotechnology, Dallas, TX, USA, sc-9168, 1: 1000), rabbit anti-CHOP (anti-GADD153) (Santa Cruz, sc-575, 1:1000), mouse anti-ATF4 (Proteintech, Rosemont, IL, USA, 10835–1-AP, 1:200), mouse anti-PCNA (Santa Cruz, sc-56, 1:1000), rabbit anti-PDX1 (Abcam, ab134150, 1:500). Alexa Fluor-conjugated chicken antibodies were used as secondary antibodies (Invitrogen).

### TUNEL staining

Pancreases were fixed overnight with 4% paraformaldehyde and embedded in paraffin. In order to detect apoptotic cells, a TUNEL assay was performed using the ApopTag fluorescein in situ apoptosis detection kit (Millipore, Massachusetts USA). Sections were triple stained with a TUNEL kit, anti-insulin antibodies, and DAPI. After being stained, the sections were observed under a confocal fluorescence microscope (BZ8100, KEYENCE, Osaka, Japan), and the ratio of TUNEL-positive cells in insulin-positive cells was calculated.

### RNA preparation and reverse transcription quantitative PCR (RT-qPCR)

Total RNA was extracted from the pancreases using a RNeasy mini kit (Qiagen, Valencia, CA, USA) and cDNA was prepared via a high-capacity cDNA Reverse Transcription kit (Applied Biosystems, Forster City, CA, USA). Samples were analyzed using the 7300 Real-Time PCR System (Applied Biosystems) using the Power SYBR Green PCR Master Mix (Applied Biosystems). The following primers were used: *β-actin*: Forward, 5′-CCTGAGCGCAAGTACTCTGTGT-3′; Reverse, 5′-GCTGATCCACATCTGCTGGAA-3′; *Atf4*: Forward, 5′- GGACAGATTGGATGTTGGAGAAAATG-3′; Reverse, 5′-GGAGATGGCCAATTGGTTCAC-3′; *Bip*: Forward, 5′-GTTTGCTGAGGAAGACAAAAAGCTC-3′; Reverse, 5′-CACTTCCATAGAGTTTGCTGATAAT-3′; *Chop*: Forward, 5′-GTCCAGCTGGGAGCTGGAAG-3′; Reverse, 5′-CTGACTGGAATCTGGAGAG-3′; *Pdx1*: Forward, 5′-ACTTGAGCGTTCCAATACGC-3′, Reverse, 5′-AGAGGGGGAACGACTCTAGG − 3′; *proinsulin* (*pro-ins)*: Forward, 5′-TCTTCTACACACACCCATGTCCC-3′; Reverse, 5′-GGTGCAGCACTGATCCAC-3′; *Xbp1*: Forward, 5′-TGGGCATCTCAAACCTGCTT-3′; Reverse, 5′-GCGTCCAGCAGGCAAGA-3′. All experiments were performed in duplicate. Results were normalized to β-actin expression level, and relative mRNA levels of the ML-diet group compared to that of the control-diet group were calculated using the 2^^(−ΔΔCt)^ method.

### Statistical analysis

Data were presented as mean ± standard error of the mean (SEM). All data were analyzed using Kaleida Graph software (version 4.5; Synergy software, Reading, PA, USA). The trapezoidal rule was used to determine the net incremental area under the curve (net AUC). The ‘*t’* test was used to determine statistically significant differences. Statistical significance was set at *P* < 0.05.

## Results

### ML-diet maintained glucose-induced insulin secretion in db/db mice

**Fig. 1:**
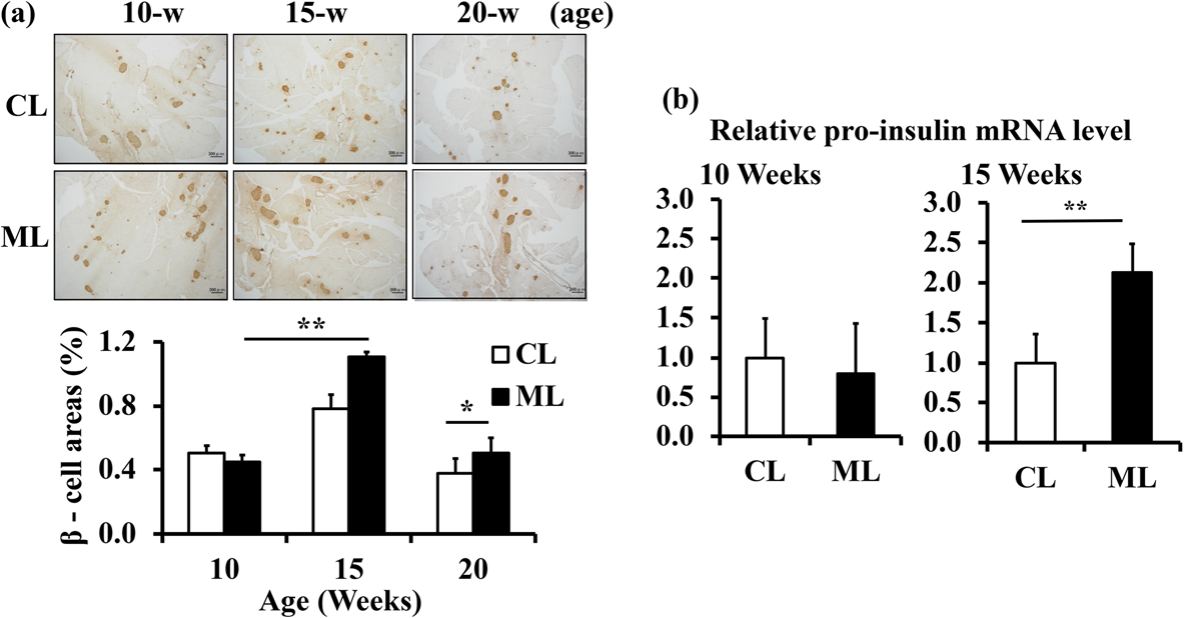
Effect of an ML-diet on the pancreases of db/db mice. **a** After being fed with an ML-diet or a control-diet from 7-w of age, the pancreases of db/db mice were collected at 10-w, 15-w, and 20-w of age, and stained with anti-insulin antibody. Scale bar, 300 μm. β-cell area (%) was calculated by dividing the insulin-positive area by the total pancreas area in randomly acquired micrographs. **b** Expression of proinsulin mRNA in pancreases of 10-w old mice and 15-w old mice was analyzed via RT-qPCR. The results are shown as relative mRNA levels of the ML-diet group to control-diet group. CL, control-diet group; ML, ML-diet group, *n* = 6. All values are mean ± SEM. *, *P* < 0.05; **, *P* < 0.01 based on a two-tailed Student’s *‘t’* test

To investigate the effects of a ML-diet on insulin level, db/db mice were fed with regular chow containing 5% (w/w) dried ML powder, from 7-weeks (7-w) of age to 20-w of age. The average body weight at 10, 15, and 20-w of age did not change between the ML-diet group and the control-diet group, suggesting that ML did not affect either the amount of food eaten or the growth of mice (Table 1). Next, immunohistological staining of pancreatic β-cells was performed using anti-insulin antibodies. The results showed that the insulin-positive area in pancreatic sections increased at 15-w compared to that at 10-w in both diet groups, while it decreased at 20-w of age (Fig. 1a). Compared to the control-diet group, pancreases of the ML-diet group showed significantly larger insulin-positive areas at 15-w and 20-w of age, suggesting that the increase in β-cell numbers was larger in the ML-diet group than that in the control-diet group. In addition, quantitative analyses indicated that mRNA expression levels of proinsulin (pro-ins) in the pancreas of mice from the ML-diet group at 15-w was significantly higher than that of the control group (Fig. 1b).

**Table 1:** Body weights of db/db mice from each diet group. Mice were fed regular chow (Control) or chow containing 5% ML (ML) from 7-w of age. Next, body weights were measured at 10-w, 15-w, and 20-w of age (*n* = 6 per group). All data are expressed as mean ± SEM. Statistically significant differences were calculated using the Student’s ‘*t*’ test

Age (weeks)	Body weight (g)		*P* value
	Control	ML	
10	40.60 ± 0.55	41.94 ± 0.73	0.18
15	45.70 ± 0.55	46.10 ± 0.85	0.70
20	50.95 ± 1.42	49.71 ± 1.01	0.51

**Fig. 2:**
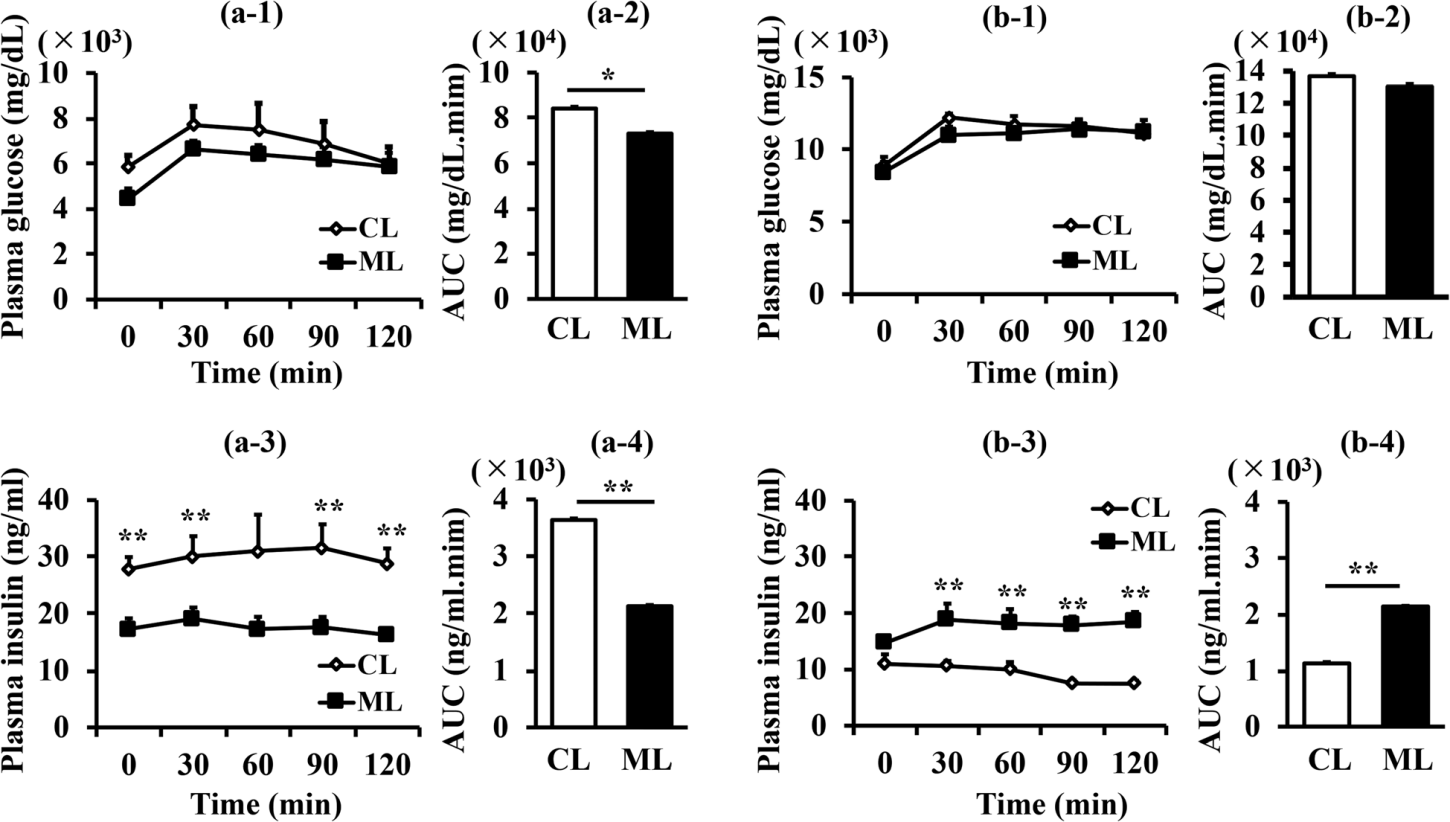
Effect of an ML-diet on glucose tolerance. Glucose tolerance was evaluated via ipGTT. After intraperitoneally injecting mice with glucose (1.5 g/kg of D-glucose), blood was drawn every 30 min, and plasma glucose and plasma insulin levels were measured. (a), 10-w old mouse; (b), 15-w old mouse; (a-1) and (b-1), Plasma glucose level (mg/dL); (a-2) and (b-2), net AUC (mg/dL.min) calculated from the data shown in (a-1) and (b-1), respectively; (a-3) and (b-3), Plasma insulin level (ng/ml); (a-4) and (b-4), net AUC (ng/mL.min) calculated from the data shown in (a-3) and (b-3), respectively. CL and white rhombus, control-diet group; ML and black square, ML-diet group; *n* = 6. Data are expressed as mean ± SEM. **, *P* < 0.01 versus control

Next, intraperitoneal glucose tolerance tests (ipGTT) were performed for 10-w old and 15-w old db/db mice as shown Fig. 2. The results showed that the ML-diet group at 10-w of age exhibited significantly decreased glucose levels compared with those of the control-diet group (Figs. 2 a-1 and a-2), while there was no significant difference between the two diet groups at 15-w of age (Figs. 2 b-1 and b-2). Interestingly, plasma insulin levels of the 10-w old and 15-w old mice in the ML-diet group were not changed (Figs. 2, black in a-3 and b-3), while plasma insulin levels in the control group were dramatically decreased (Figs. 2, white in a-3 and b-3). These results demonstrated that ML-intake maintained β-cell function.

### The effect of ML-diet on ER stress in the pancreas

**Fig. 3:**
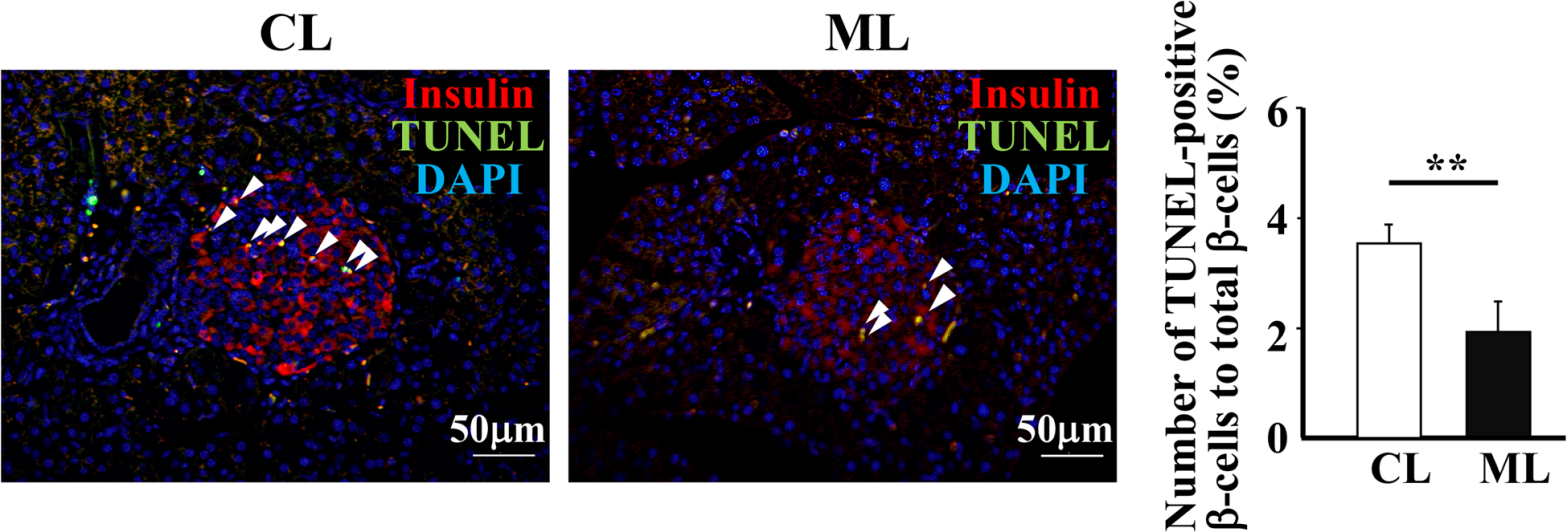
The effect of an ML-diet on cell death in the pancreases of db/db mice. Pancreatic sections from 15-old db/db mice were stained using the TUNEL method (green), anti-insulin antibodies (red) and DAPI (blue). White triangles indicate TUNEL-positive cells. CL, control-diet group; ML, ML-diet group. Scale bar: 50 μm. The ratio of the number of TUNEL-positive β-cells to total β-cell numbers is shown in the right panel. **, *P* < 0.01 versus control

**Fig. 4:**
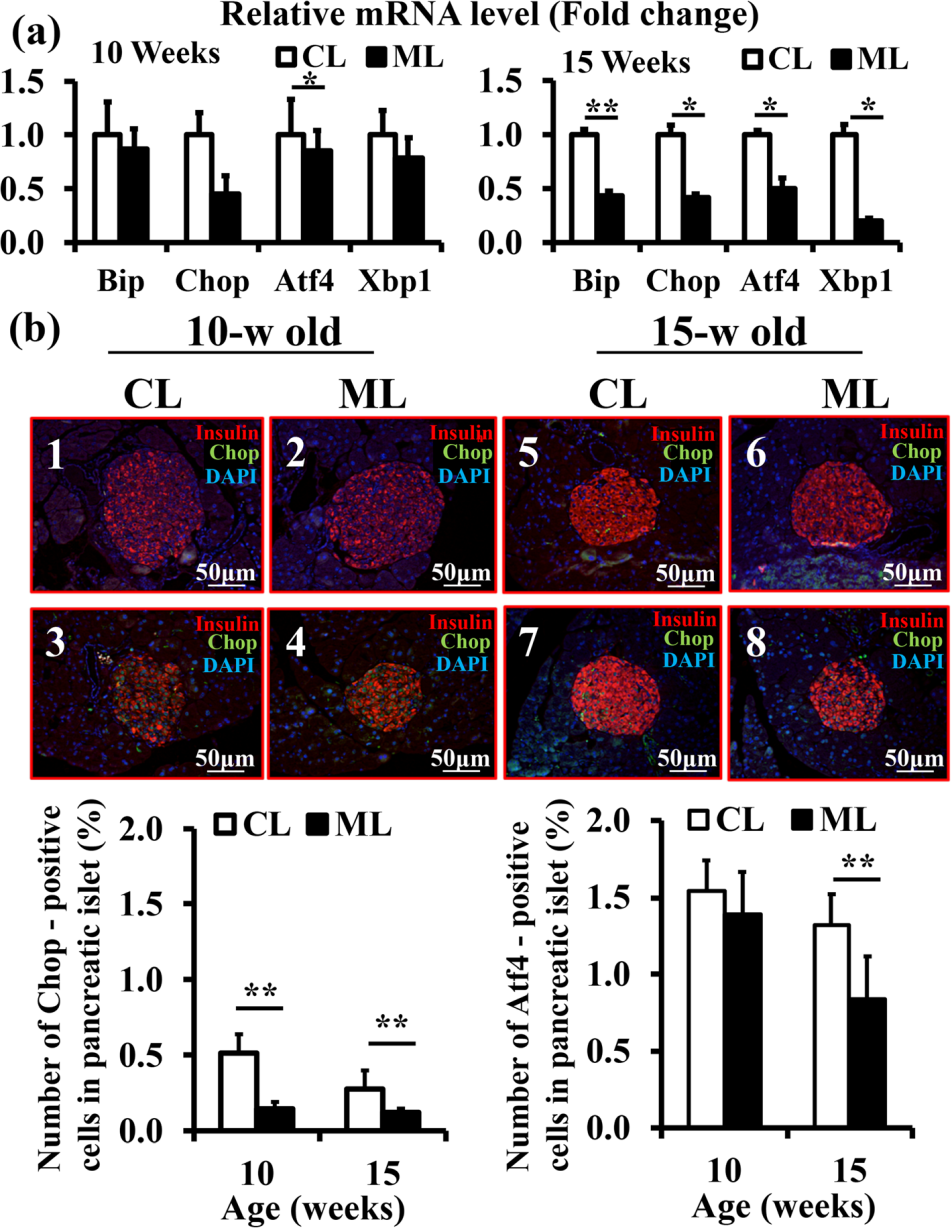
The effect of an ML-diet on ER stress in β-cells of db/db mice. **a** mRNA expression of ER stress markers in pancreases of 10-w old mice and 15-w old mice were analyzed via RT-qPCR. The results are shown as relative mRNA levels of the ML-diet group to the control-diet group. **b** Immunohistological analyses of CHOP and ATF4. Pancreases of 10-w old and 15-w old mice were stained with anti-CHOP or anti-ATF4 antibodies (green), anti-insulin antibodies (red), and DAPI (blue), observed under a confocal fluorescence microscope. 1–4, pancreas from a 10-w old mouse; 5–8, pancreas from a 15-w old mouse; 1, 2, 5, 6; stained with anti-CHOP antibodies; 3, 4, 7, 8; stained with anti-ATF4 antibodies. The ratio of the number of CHOP or ATF4-positive β-cells to total β-cells are shown in the lower panel; *n* = 6. CL and white bar, control group; ML and black bar, ML-diet group. All data are expressed as mean ± SEM. *, *P* < 0.05; **, *P* < 0.01

ER stress in β-cells, accompanied by the secretion of mature insulin, is involved in the progress of T2DM. To reveal mechanisms underlying significantly large increases of β-cell mass in the ML-diet group, the effects of ML-diet suppression on cell death and ER stress in the pancreas were examined. TUNEL-positive β-cells in the pancreases of the ML-diet group were significantly reduced compared to those in the control-diet group, suggesting that ML intake suppressed apoptosis of β-cells (Fig. 3). Next, mRNA levels of various ER stress markers in the pancreases were quantified via RT-qPCR. The results showed that the mRNA level of *Chop* at 10-w of age and those of ER stress markers, *Bip*, *Chop*, *Atf4,* and *Xbp1,* at 15-w of age, were significantly reduced by the ML-diet compared to those of the control-diet (Fig. 4a). In addition, immunohistochemical analyses of islets demonstrated that expression of both CHOP and ATF4 proteins were also decreased in the ML-diet group than that in the control diet group (Fig. 4b). Considered together, these data suggested that ML-diet suppressed β-cell apoptosis by reducing ER stress, thereby maintaining β-cell function.

### The effect of ML-diet on β-cell proliferation

**Fig. 5:**
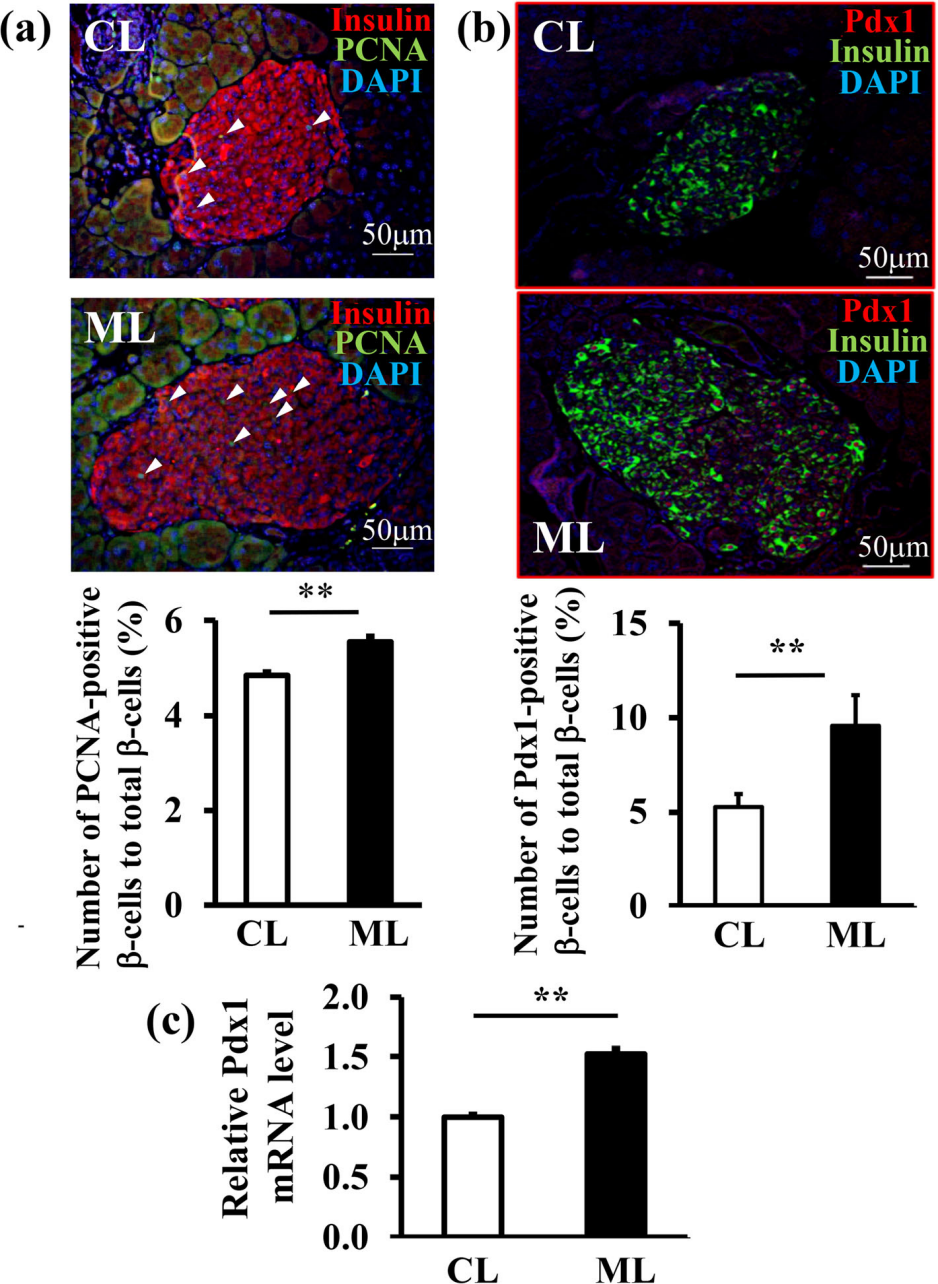
The effect of an ML-diet on the proliferation of β-cells in db/db mice. **a** Representative fluorescence image of a pancreas from a 10-w old db/db mouse stained with anti-PCNA antibodies (green), anti-insulin antibodies (red), and DAPI (blue). White triangles indicate PCNA-positive cells. Scale bar: 50 μm. The ratio of the number of PCNA-positive β-cells to total β-cell number is shown in the graph (right panel). **b** A representative fluorescence image of a pancreas from a 15-w old db/db mouse stained with anti-PDX1 antibodies (red), anti-insulin antibodies (green) and DAPI (blue). The ratio of the number of PDX1-positive cells to the total β-cell number is shown in the graph (right panel). **c** Expression levels of *Pdx1* mRNA in the pancreas of a 15-w old db/db mouse were analyzed via RT-qPCR. The relative *Pdx1* mRNA level of the ML group to the control group is shown. CL, control-diet group; ML, ML-diet group, *n* = 6. All data are expressed as mean ± SEM. **, *P* < 0.01 versus control

β-cell proliferation was evaluated via immunohistological staining with anti-PCNA (proliferating cell nuclear antigen) antibodies. PCNA-positive cells were significantly increased (*P* < 0.01) in the pancreases of the ML-diet group compared to those in the control-diet group (Fig. 5a). Next, the expression of pancreatic duodenum homeobox-1 (*Pdx1*), a marker of pancreatic bud cells in the pancreas, in 15-w old mice, was examined using immunostaining and RT-qPCR. Results indicated that the expression of both protein and mRNA of *Pdx1* in the ML-diet group was significantly increased compared to that in the control-diet group (Figs. 5b and c). These results suggested that the ML-diet may induce pancreatic β-cell proliferation.

## Discussion

Diabetes mellitus is a common metabolic disorder characterized by the destruction of pancreatic β-cells or diminished secretion and function of insulin [20]. In order to elucidate mechanisms for the enhanced glucose tolerance effects of ML, the current study analyzed the effect of oral ML administration from 7 weeks onwards on pancreatic β-cells, using db/db mice. The insulin-positive area was increased at 15-w of age compared with that of 10-w in both diet groups, while it was decreased at 20-w of age (Fig. 1a). This age-dependent change in β-cell mass suggested that an increase in β-cell mass occurred at the compensation stage but decreased subsequently at the decompensation stage. Observation of the β-cell area of 15-w and 20-w old mice demonstrated that ML administration has the potential to maintain β-cells (Fig. 1a). The β-cell area in the pancreas of 15-w old ML-diet mice was larger than that of the control-diet group, suggesting that an ML diet may enhance the increase in β-cell mass at the compensation stage. This effect of an ML-diet may help to maintain β-cell mass at the decompensation stage. These effects on β-cells are supported by ipGTT results (Fig. 2).

To reveal the mechanism that maintains β-cell mass via an ML-diet, cell death and proliferation were analyzed. Results indicated that an ML-diet reduced apoptotic death of β-cells compared with that of the control group (Fig. 3) and suppressed the expression of genes encoding ER stress markers in the pancreas (Fig. 4). These finding suggested that cell death was suppressed by the reduction of ER stress due to ML-intake. Furthermore, an ML-diet enhanced proliferation of β-cells (Fig. 5). Thus, demonstrating that an ML-diet may maintain the β-cell mass via both enhancement of proliferation at the compensation stage and reduction of cell death at the decompensation stage in db/db mice.

Many studies have reported that the intake of some natural plant products improves insulin resistance and suppresses hyperglycemia, without affecting the expression of the BiP/GRP78 chaperone in response to ER stress. Kim et al., reported that a traditional Korean medicine known as Chinese mulberry (*Cudrania tricuspidata*), which is in the *Moraceae* family, suppressed expression of ER stress marker mRNAs, such as *Chop* and *Atf4,* in the liver of db/db mice, while no significant change was observed in the expression of *Bip/grp78* [23]. By contrast, the current study demonstrated that an ML-diet suppressed the expression of *Bip*, a chaperone, in the pancreas. Another study reported that ML supplementation suppressed the expression of *bip/grp78* in the myocardia of experimental autoimmune myocarditis rats [24]. These findings suggest that ML may possess a special capability for bioactivity among herbs that reduce ER stress.

Furthermore, other studies have indicated that quercetin suppressed the expression of *bip/grp78* in the intestinal human colon adenocarcinoma cell line LS180 [25], and also suppressed the expression of *bip/grp78* and *Chop,* induced via tunicamycin in endothelial cells [26]. ML is known to contain quercetin 3-*O*-glucoside and kaempherol 3-*O*-glucoside [27, 28]. Furthermore, Enkhmaa et al. identified quercetin 3-(*6*-malonylglucoside) (Q3MG) as a main antioxidant contained in ML, and showed that Q3MG attenuated the development of atherosclerotic lesions is mouse models [29], as opposed to quercetin, which did not. Quercetin is minimally absorbed by the intestine, and therefore flavonoid glycoconjugates may be responsible for the maintenance of β-cell mass by ML intake. However, since these flavonoids are also contained in various other plants, further studies may be needed to clarify the specific bioactivity of ML.

Our experiments demonstrated that an ML-diet promoted β-cell proliferation and enhanced *Pdx1* expression (Fig. 5). A previous study indicated that impaired activation of the insulin signaling pathway, P13K and protein kinase B (PKB/Akt) signaling, suppressed *Pdx1* expression [30], leading to the dysfunction of mature β-cells [31]. Other studies have demonstrated that regeneration of β-cells is mediated by *Pdx1* expression. For example, spontaneous diabetes in a zebrafish model recovered rapidly following chemical treatment, due to PCNA-Pdx1 positive cells differentiating into β-cells [32]. These reports indicate that upregulation of *Pdx1* expression in db/db mice fed ML may induce β-cell regeneration.

Li et al. administered a mixture of polysaccharide and DNJ, a α-glucosidase inhibitor contained in ML, to mice in whom diabetes was induced via alloxan. They showed that insulin expression was upregulated via increased *Pdx1* expression in the pancreas [33]. Similarly, in an in vitro model, dysfunctional β-cells induced by fructose showed a significant increase in cell proliferation by incubation with quercetin, suggesting that quercetin may prevent β-cell dysfunction [34]. In addition, substances such as charcoal were found in the flowering stages of ML. Hu et al. treated RIN-5F cells, rat pancreatic β-cells stimulated by high glucose, with charcoal extracted from dried flower buds (*Cleistocalyx operculatus*). The results showed that dried flower buds protected cells from damage by suppressing nitric oxide (NO) production and increasing the mRNA expression of *pro-insulin* and *Pdx1* [35]. These reports substantiate the results of the present study, confirming that ML-intake increased *Pdx1* expression, which in turn promoted β-cell proliferation.

## Conclusions

**Fig. 6:**
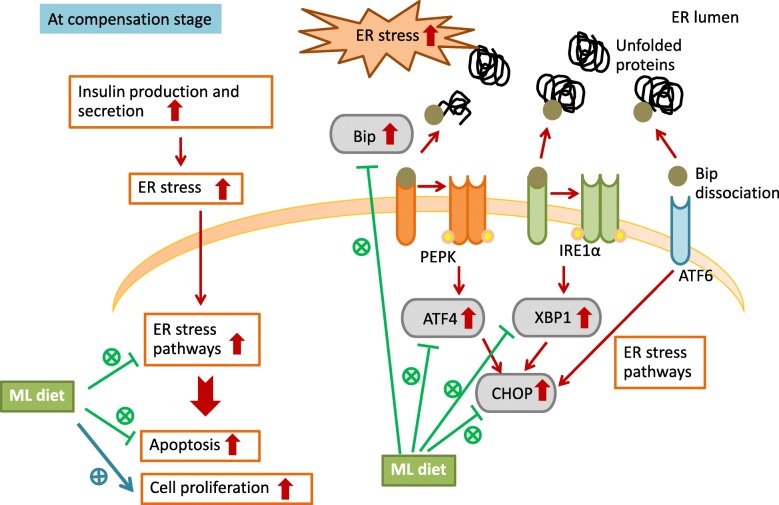
Hypothetical scheme representing the protective effect of an ML-diet on β-cells from dysfunction induced by ER stress

In conclusion, this study indicated that ML-intake maintains β-cell mass via both mechanisms, induction of β-cell proliferation and suppression of β-cell apoptosis, in mouse obesity/T2DM models. It was also shown that the suppression of β-cell apoptosis was caused by reduced ER stress. Moreover, ML-intake enhanced *Pdx1* expression in the pancreas, thereby restoring β-cell function as proposed in Fig. 6. Considered together, these findings suggested that ML contains several bioactive substances which exhibit complementary potency and may be useful for developing an effective treatment regimen for diabetes mellitus.

## Supplementary information

**Additional file 1.**

## Data Availability

The datasets used and/or analyzed during the current study available from the corresponding author on a reasonable request.

## Abbreviations

PI3K: Phosphatidylinositol-3-kinase
PCNA: Proliferating cell nuclear antigen
ROS: Reactive oxygen species
TUNEL: Terminal deoxynucleotidyl transferase mediated dUTP nick end labeling
BiP: Immunoglobulin heavy-chain binding protein
GRP78: Glucose related protein 78
IRE1: Inositol requiring enzyme 1
PERK: PKR like endoplasmic reticulum kinase
ATF6: Activating transcription factor 6
*ATF4*: *Activating transcription factor 4*
*XBP1*: *X-box binding protein1*
*CHOP*: *C/EBP homologous protein*
PI3K: Phosphatidylinositol-3-kinase
*Pdx1*: *Pancreatic duodenal homeobox1*
DNJ: 1-deoxynojirimycin
Q3MG: Quercetin 3-(*6*-malonylglucoside)
RT-qPCR: Reverse transcription quantitative polymerase chain reaction
ipGTT: Intraperitoneal glucose tolerance tests
AUC: Area under the curve
