# Supplementary material for: Administration of mulberry leaves maintains pancreatic β-cell mass in obese/type 2 diabetes mellitus mouse model
Source: BMC Complement Med Ther. 2020 May 6;20:136. doi: 10.1186/s12906-020-02933-4 (PMC7201661; doi:10.1186/s12906-020-02933-4)
Supplement: Supplementary file 1 — Additional file 1. [file 12906_2020_2933_MOESM1_ESM.pptx]

## Slide 1
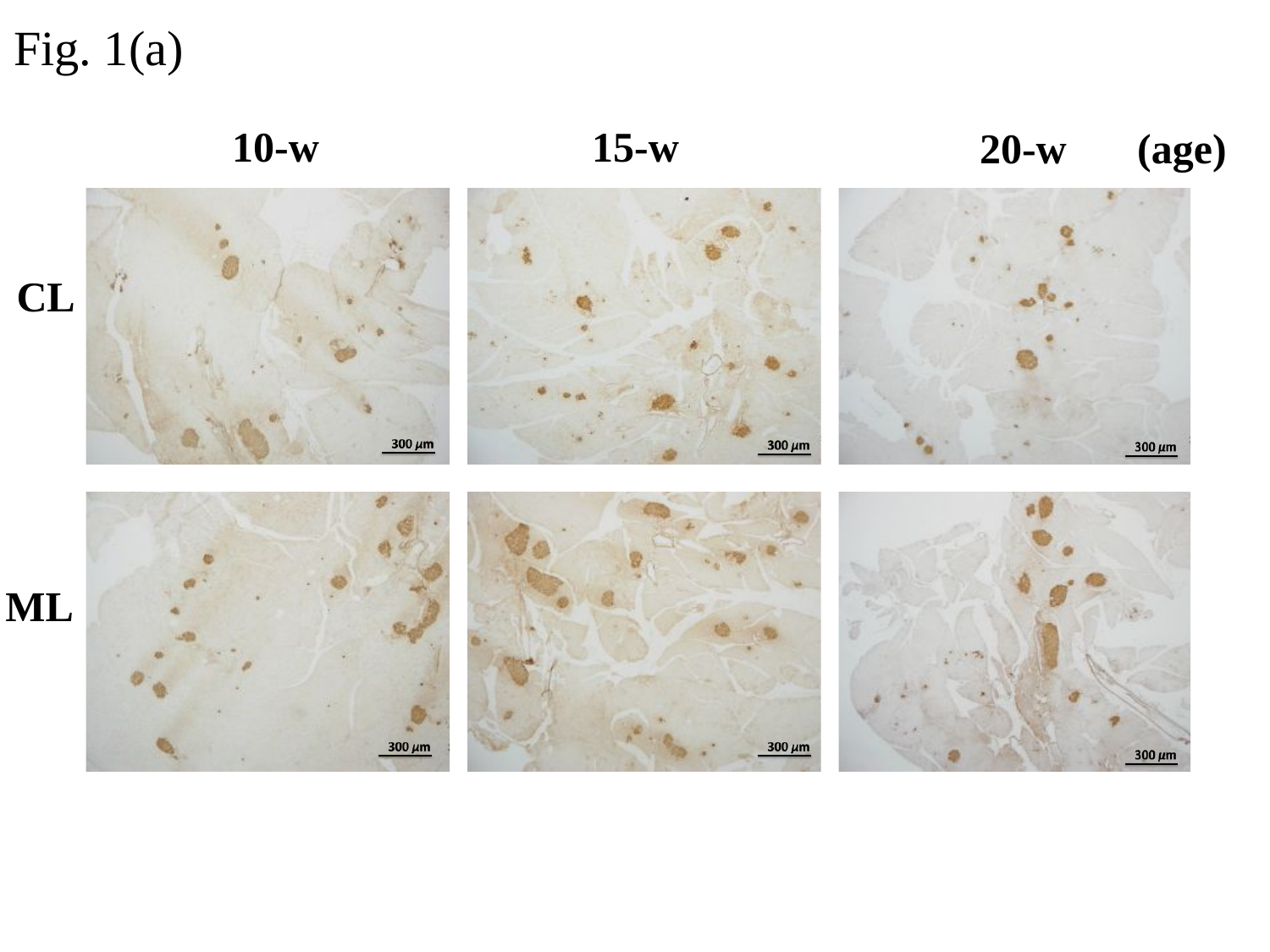

Fig. 1(a)
10-w
15-w
20-w
(age)
CL
ML

## Slide 2
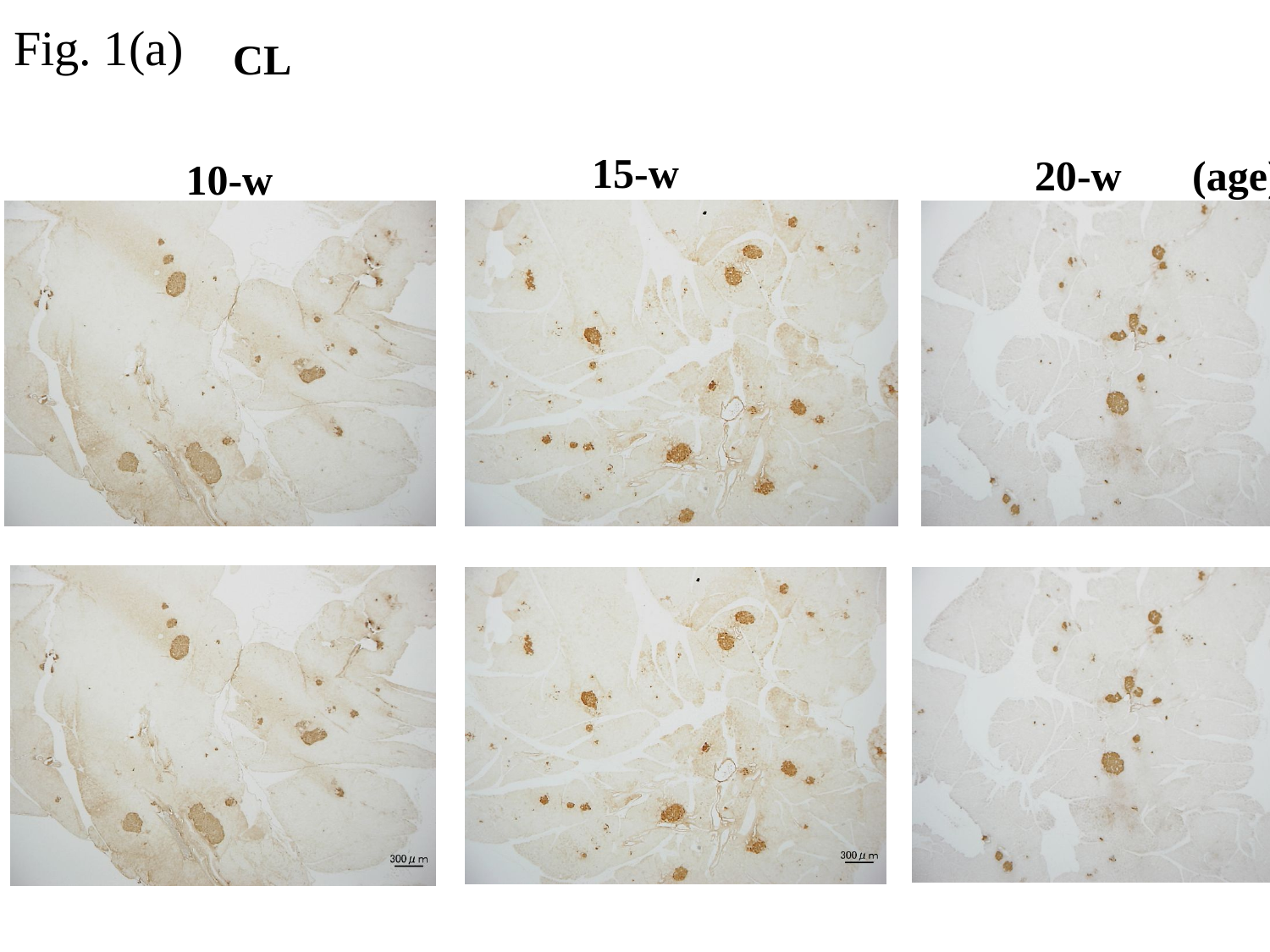

Fig. 1(a)
CL
15-w
20-w
(age)
From Mr. OKADA file.
10-w

## Slide 3
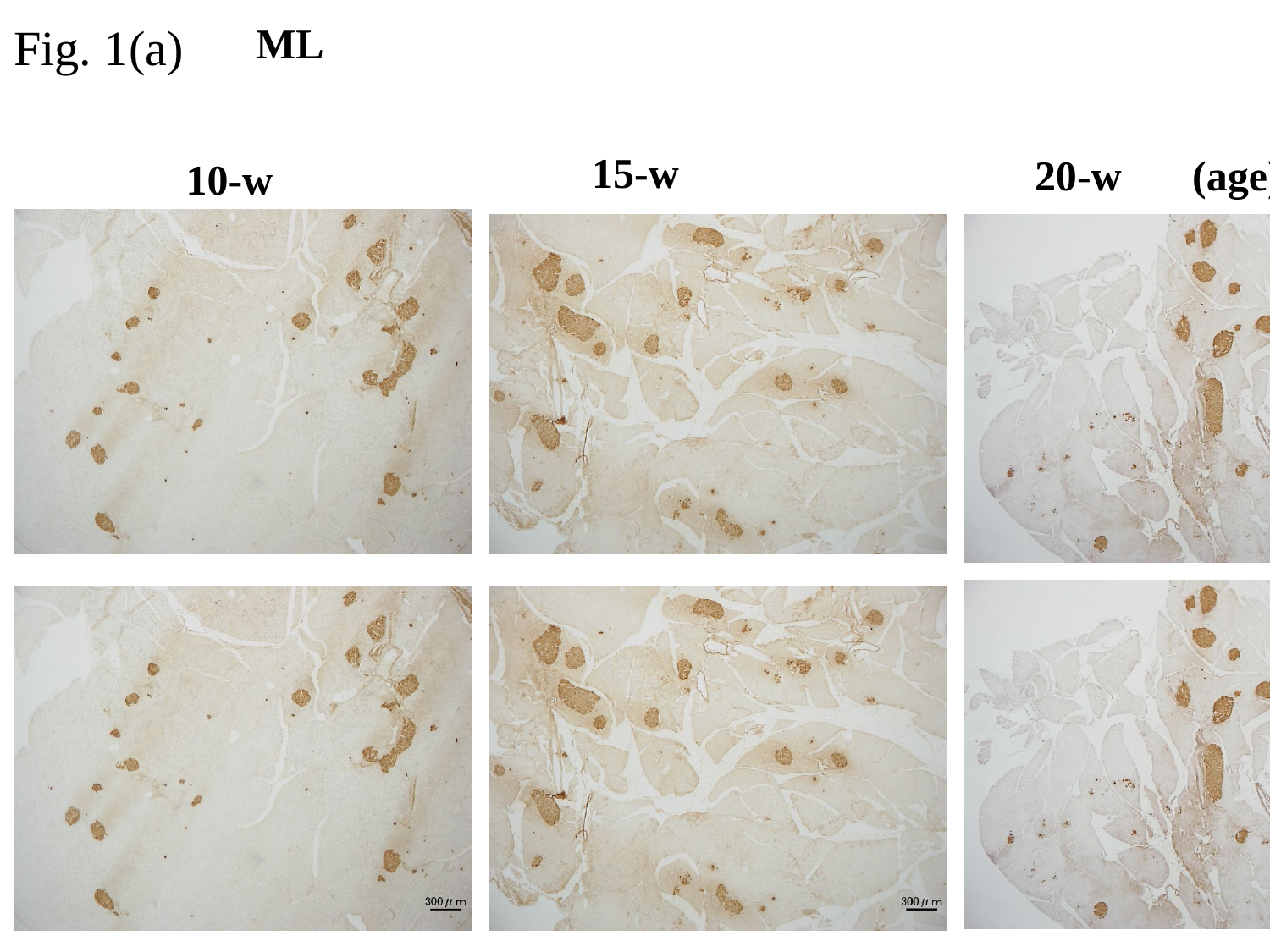

Fig. 1(a)
ML
15-w
20-w
(age)
10-w

## Slide 4
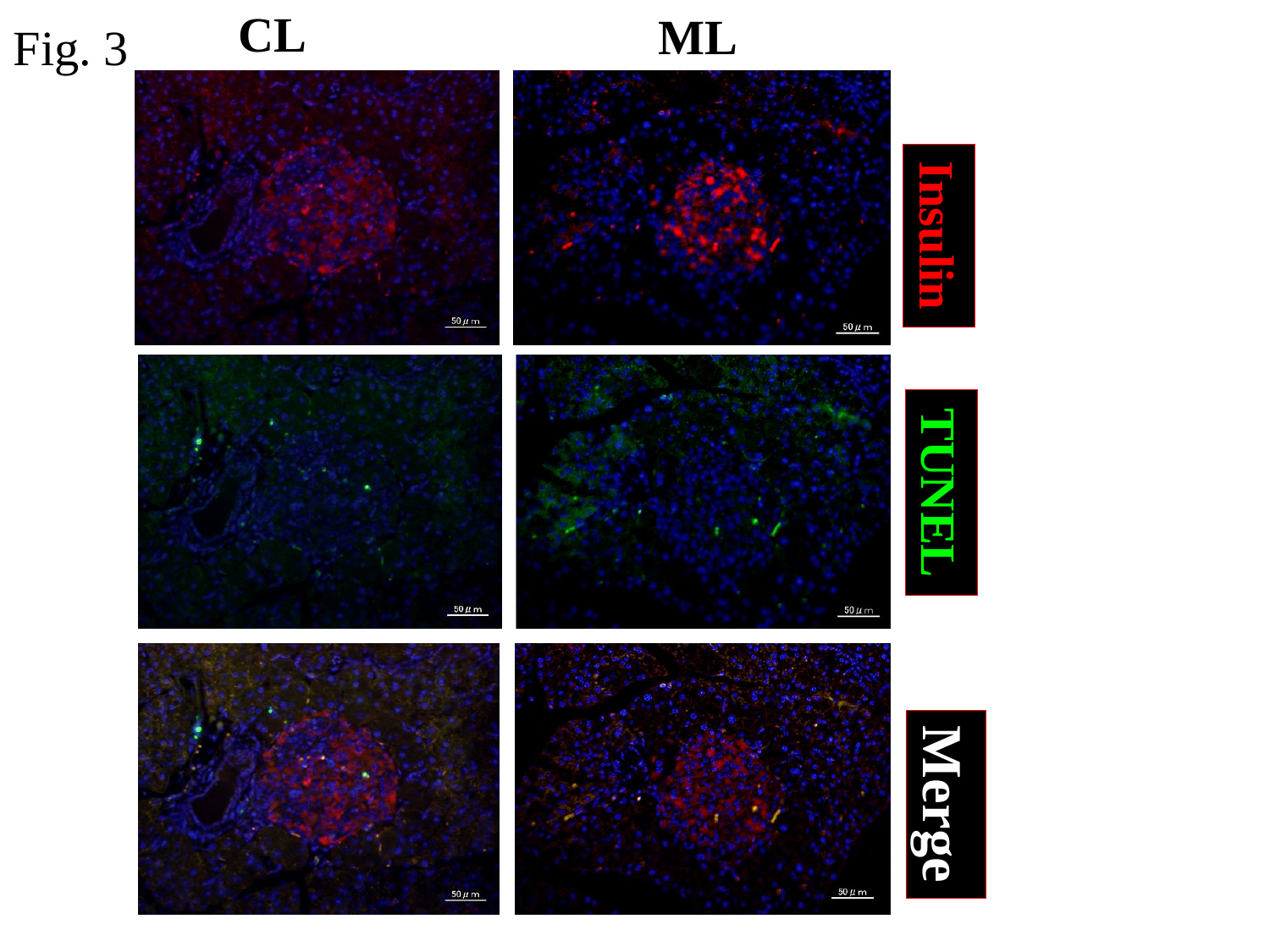

CL
ML
Fig. 3
Insulin
TUNEL
Merge

## Slide 5
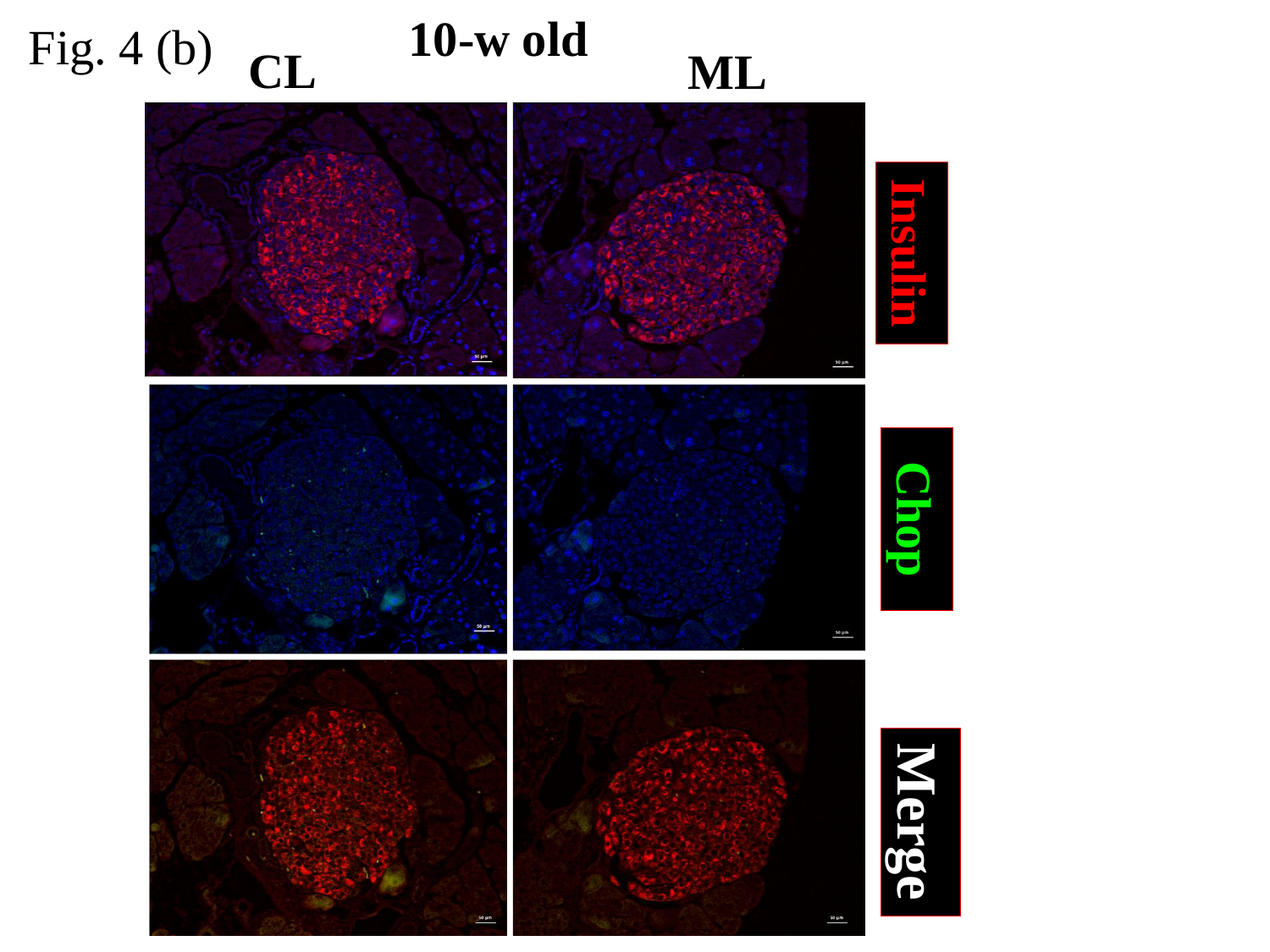

10-w old
Fig. 4 (b)
CL
ML
Insulin
Chop
Merge

## Slide 6
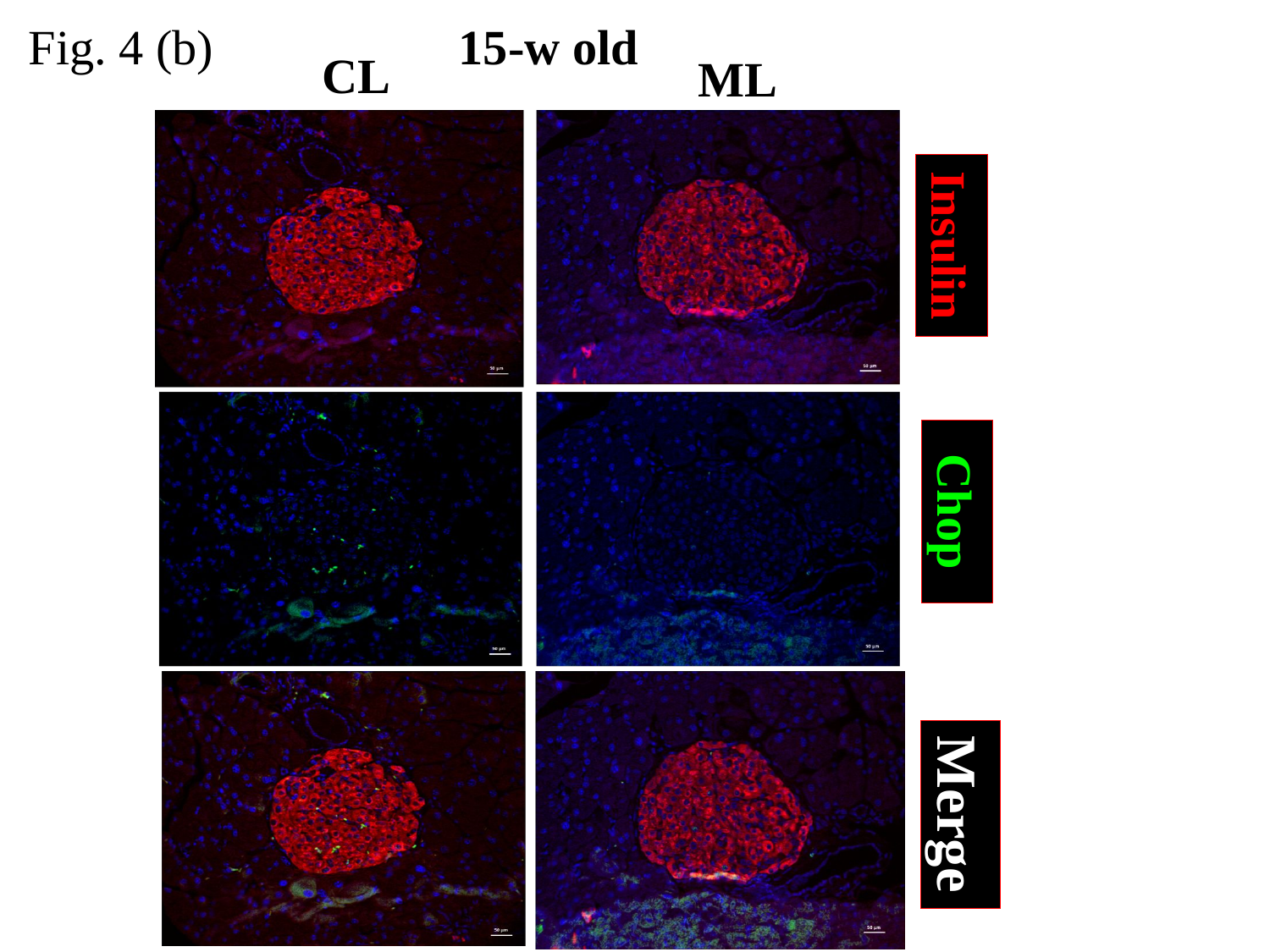

Fig. 4 (b)
15-w old
CL
ML
Insulin
Chop
Merge

## Slide 7
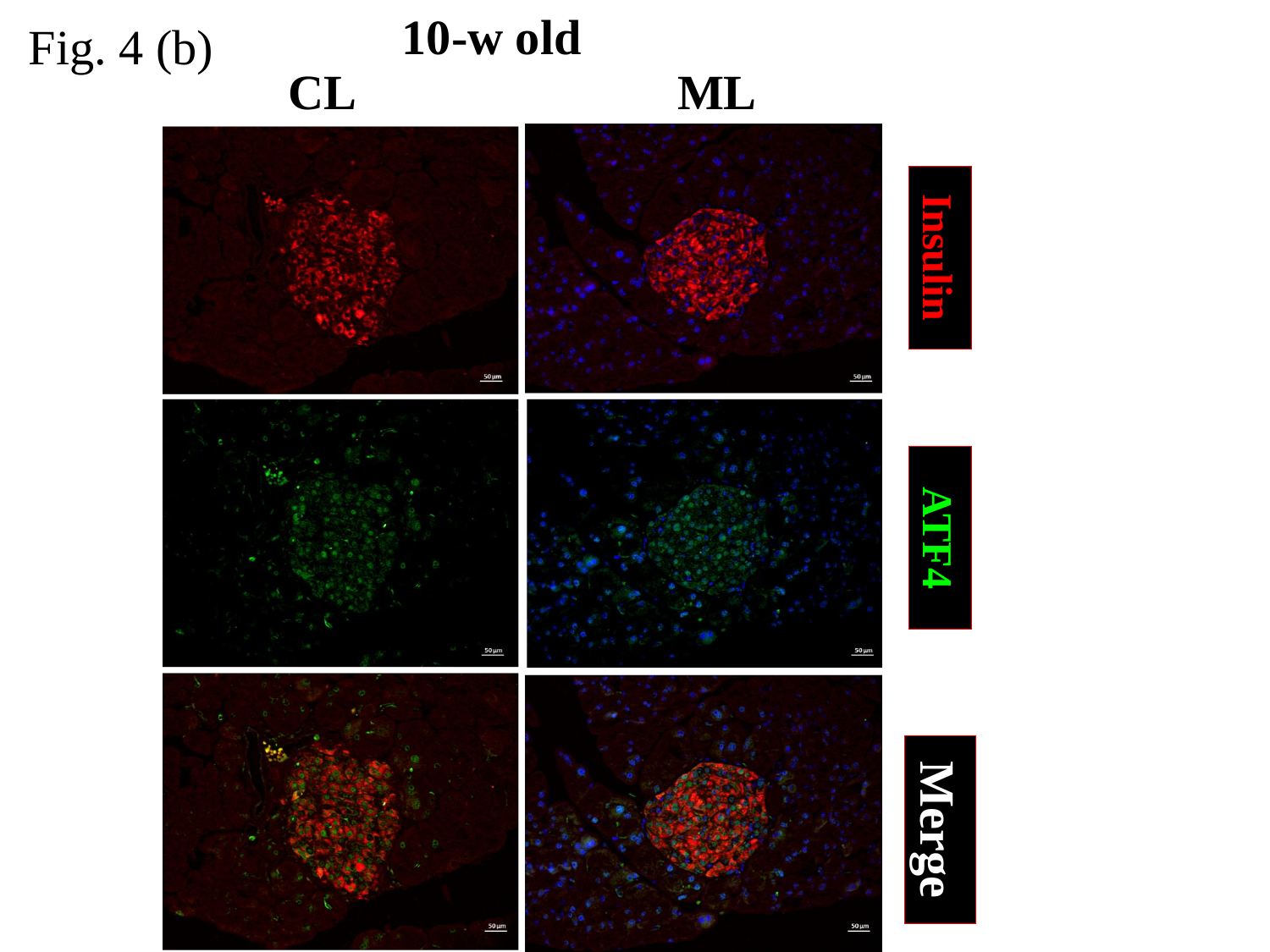

10-w old
Fig. 4 (b)
CL
ML
Insulin
ATF4
Merge

## Slide 8
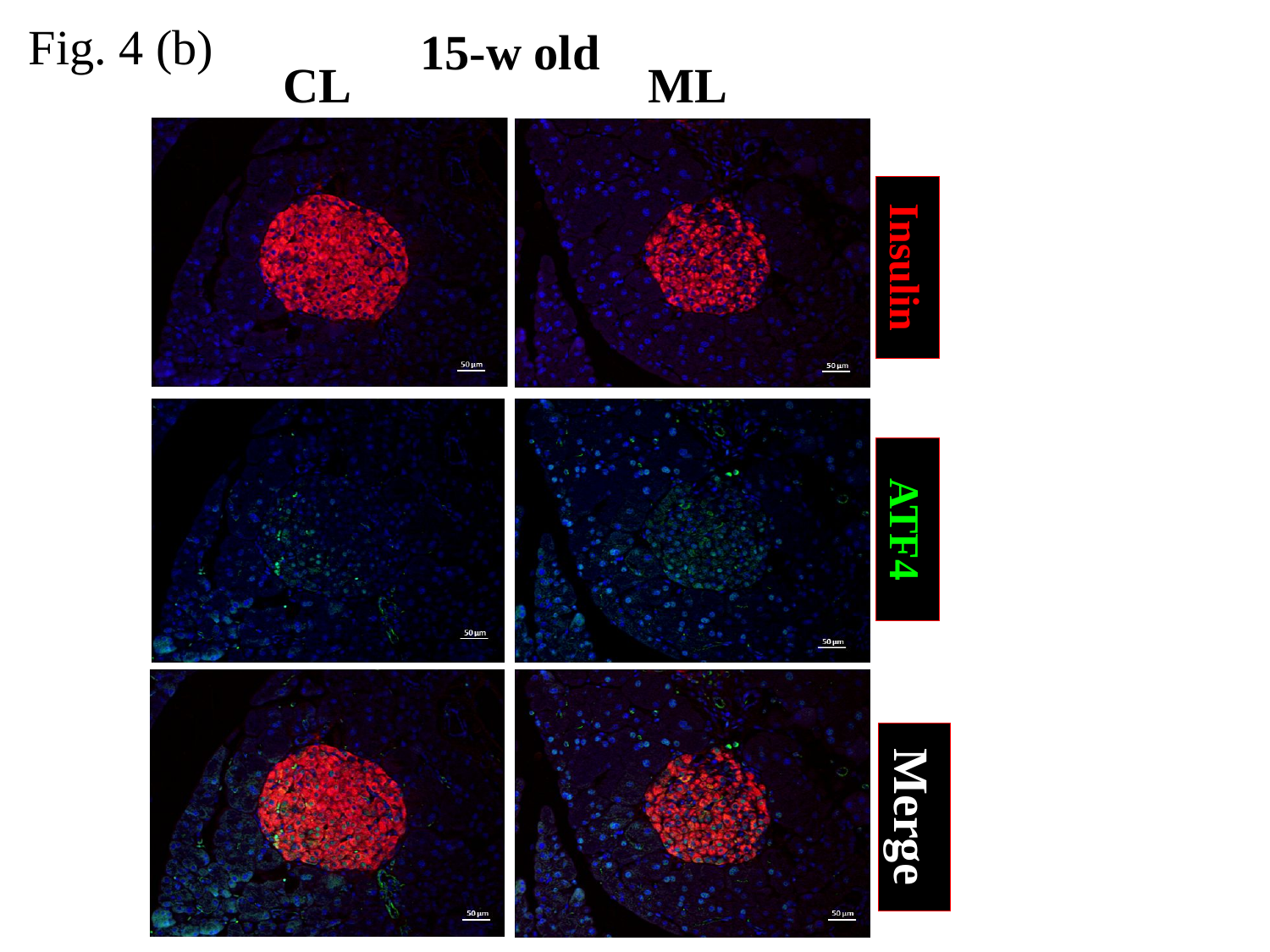

Fig. 4 (b)
15-w old
CL
ML
Insulin
ATF4
Merge

## Slide 9
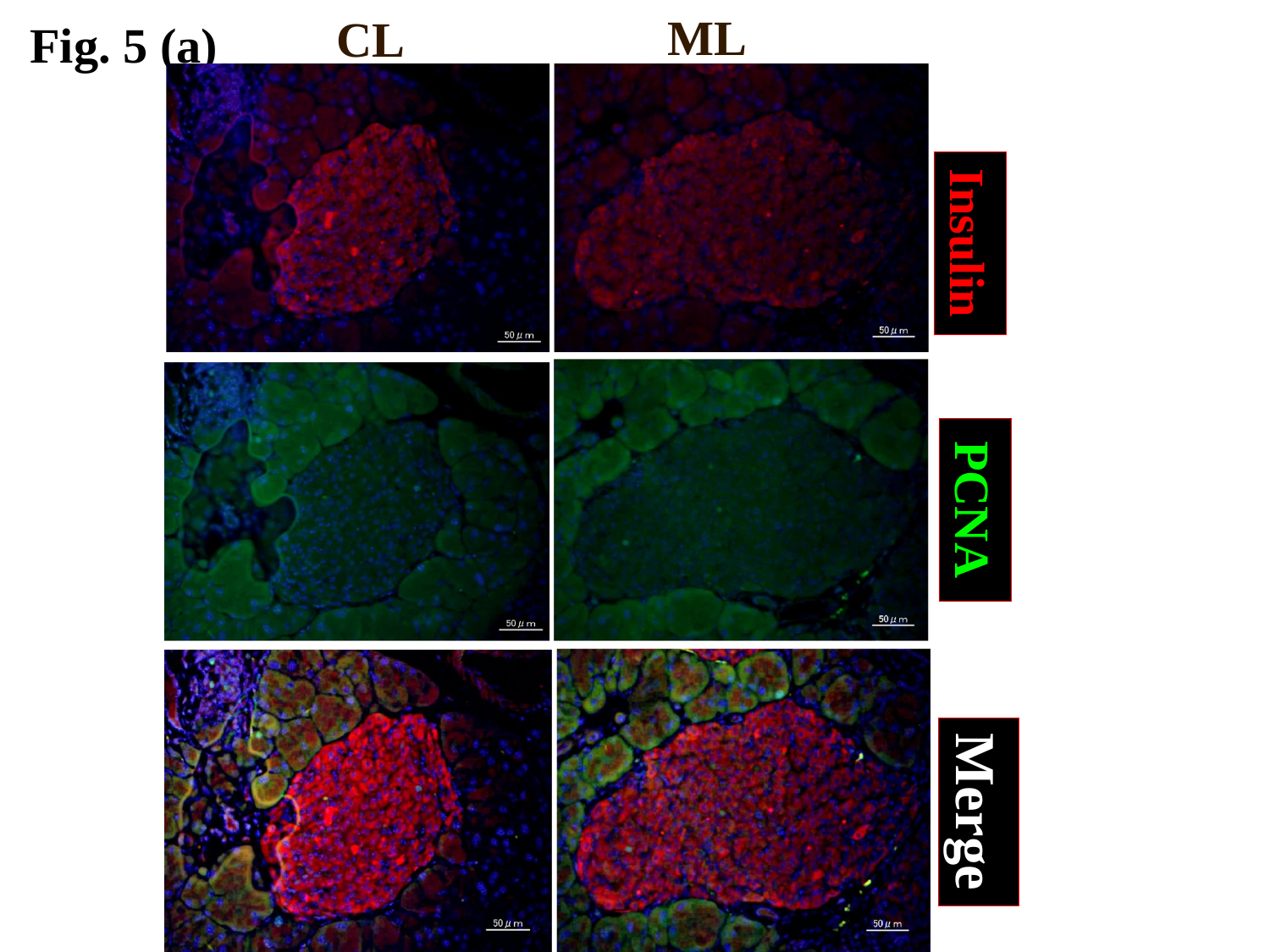

ML
CL
Fig. 5 (a)
Insulin
PCNA
Merge

## Slide 10
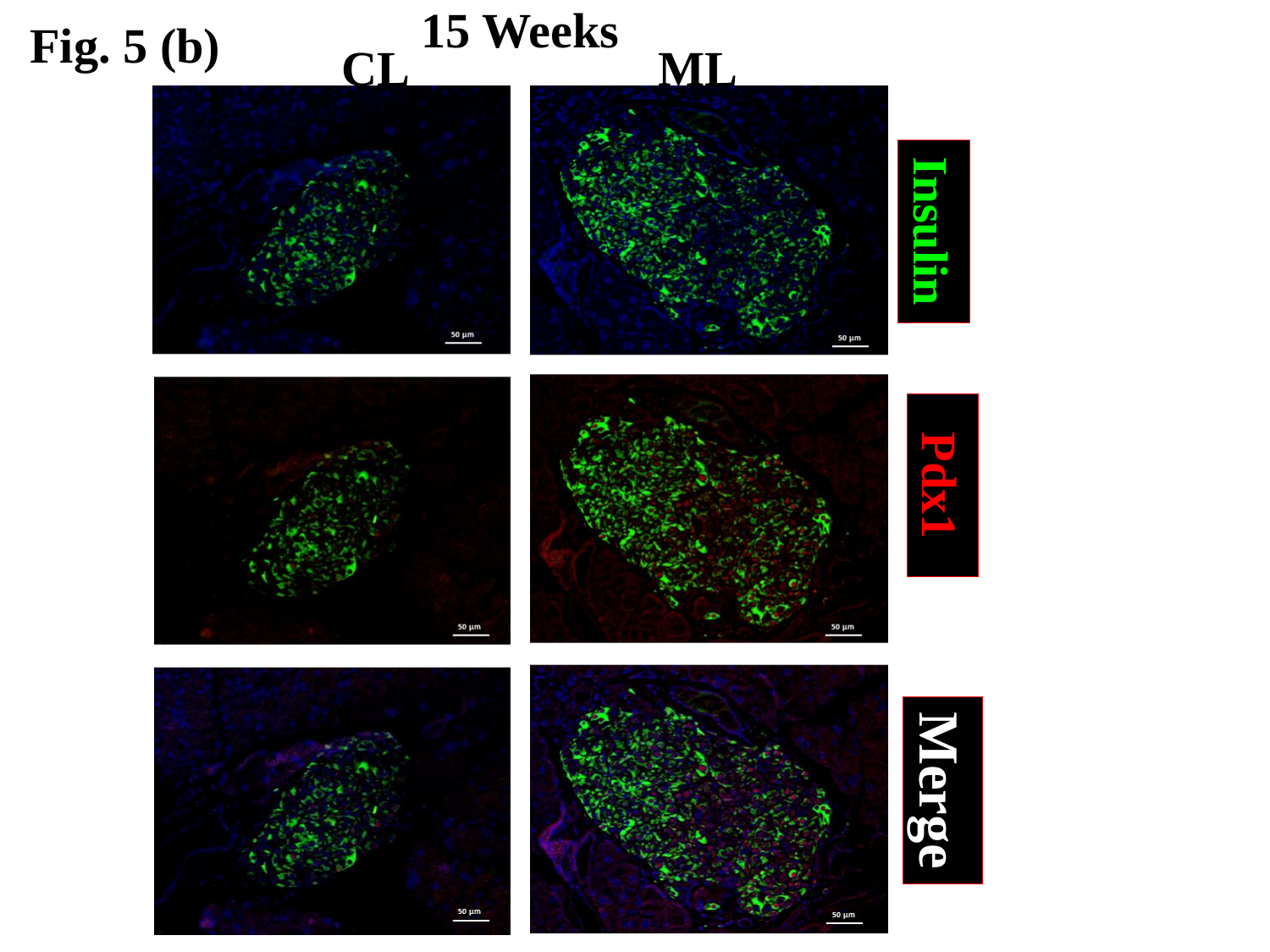

15 Weeks
Fig. 5 (b)
CL
ML
Insulin
Pdx1
Merge

## Slide 11
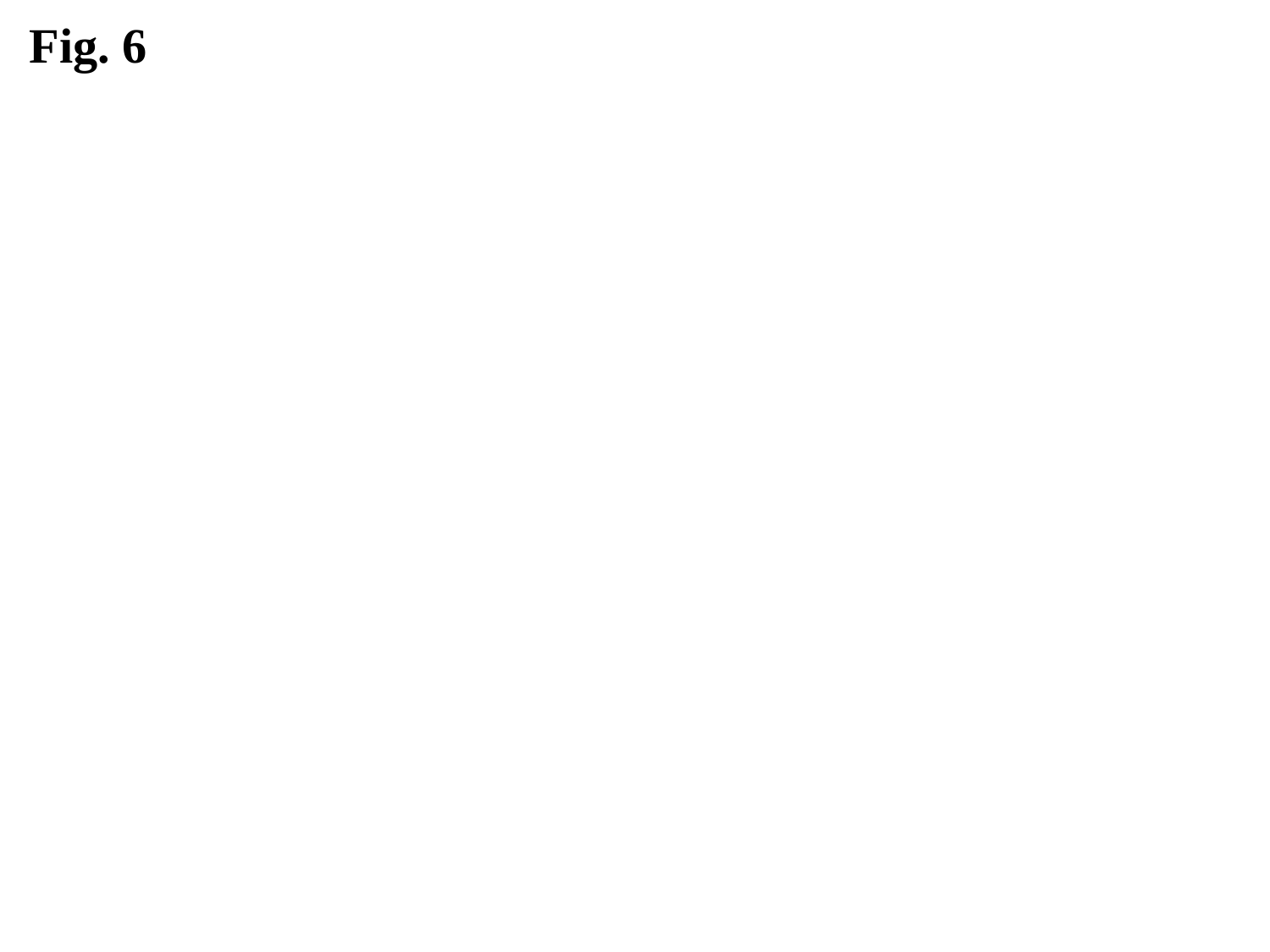

Fig. 6
